# Effects of hydroxypropyl starch on intestinal health and transcriptome of geese

**DOI:** 10.1038/s41598-025-96020-8

**Published:** 2025-04-10

**Authors:** Pengfei Ye, Wenquan Liu, Xiaotong Tang, Mengxue Liu, Jingfan Han, Xiaoxue Wang, Jie Zhu, Xiaorong He, Xueqi Zhu, Mixia Cao, Lei Zhao, Qingchang Ren

**Affiliations:** 1 College of Animal Science, Anhui Science and Technology University, Chuzhou, 239000 China; 2 Anhui Province Key Laboratory of Animal Nutritional Regulation and Health, Chuzhou, 233100 China

**Keywords:** Goose, Hydroxypropyl starch, Intestinal health, Uric acid metabolism, Transcriptome analysis, Genetics, Molecular biology, Zoology

## Abstract

In recent years, gout resulting from uric acid metabolism disorders has led to significant economic losses in goose production. The intestine is a vital organ crucial for uric acid metabolism. Hydroxypropyl starch (HPS) is a resistant starch modified from natural starch, which can enhance intestinal health as a dietary ingredient fiber. In this study, 240 30-day-old Yangzhou geese with similar body weights were divided into three groups: The control group (CG) received a basal diet + 5% corn starch; the hydroxypropyl starch group (HPS) and the sodium urate group (SU) were given a basal diet + 5% hydroxypropyl starch. The experiment lasted for 21 days, and the SU group was administered 30 mg of sodium urate per day during the last 4 days of the study. The results indicated that the level of uric acid in the HPS group was 56.6 µmol/L, significantly lower than that in the CG group (70.8 µmol/L) and the SU group (129.7 µmol/L). The morphological findings revealed that the ileum of the CG group and the SU group exhibited varying degrees of damage, while the HPS group maintained complete structure. The villus height and the ratio of villus height to crypt depth in the HPS group were significantly higher compared to those in the CG and SU groups, while the crypt depth was significantly lower than that in the SU group. A total of 1462 differentially expressed genes (DEGs) were identified at the transcriptome level. GO and KEGG functional enrichment analyses indicated that the DEGs were significantly enriched in the Brush border membrane, Brush border, PPAR signaling pathway, PI3K-Akt signaling pathway, and other related processes. Subsequent analysis revealed that HPS up-regulated the expression of genes associated with intestinal function (such as SLC5A12 and SLC5A8), structure (including NR5A2, IPMK), and uric acid metabolism (PDZK1). The accuracy and reliability of transcriptome sequencing data were confirmed by RT-qPCR. In this study, we systematically demonstrated that HPS can improve intestinal morphology and reduce serum uric acid levels, emphasizing its potential as a dietary supplement for geese.

**Supplementary Information:**

The online version contains supplementary material available at 10.1038/s41598-025-96020-8.

## Introduction

Factors including viral infection, nutritional imbalance, or impaired kidney function can result in uric acid metabolic disorder in poultry, leading to avian gout^1^. Uric acid is primarily produced in the liver, intestines, and blood vessels endothelium^2^. The intestinal tract is the primary organ for the extrarenal metabolism of uric acid^3^, and approximately 1/3 of the uric acid produced per day is metabolized through the intestinal tract^4^.

The small intestine is the body’s most essential digestive organ. As a part of the small intestine, the ileum contains a wealth of intestinal microorganisms^5^. Research shows some bacteria can directly participate in uric acid metabolism^6^. The intestinal flora of gout patients is significantly different from that of healthy people^7^ and leads to changes in intestinal structure^8^. The complete intestinal structure underlies the function of uric acid transporters. Previous studies have demonstrated that ABCG2 serves as a high-capacity uric acid transporter, with abundant expression found in the brush border membrane of small intestinal epithelial cells, playing a significant role in uric acid excretion ^9^. SLC16A9 also has similar functions ^10^, while SLC2A9 is involved in uric acid reabsorption ^11^. Additionally, some proteins, such as PDZK1 and CARMIL MAF, are indirectly involved in regulating serum uric acid levels^12^.

Resistant starch (RS) refers to starch that cannot be directly digested and absorbed by the body. As a type of dietary fiber, it positively affects intestinal conditions^13^. Microorganisms can ferment it as a substrate to produce short-chain fatty acids (SCFAs), which provide energy for epithelial cells, aiding in the metabolism of uric acid and enhancing the integrity of the intestinal epithelial barrier^14,15^. Hydroxypropyl starch (HPS) is a resistant starch that is artificially modified from natural starch^16^. Studies indicate that dietary fiber can lower serum uric acid levels by decreasing the absorption of adenine from food^17,18^. Previous research has shown that HPS leads to significant changes in intestinal flora^19^ and may influence plasma cholesterol concentration by producing propionic acid^20^. However, there is currently no research on the direct effect of HPS on uric acid metabolism.

In this study, we evaluated the effect of HPS on geese through intestinal morphology and serum uric acid levels, while also investigating the molecular mechanism of HPS on intestinal health and uric acid metabolism in geese using transcriptomics. This research provides a scientific basis for HPS as a feed additive for geese.

## Results

### Serum uric acid levels

**Fig. 1:**
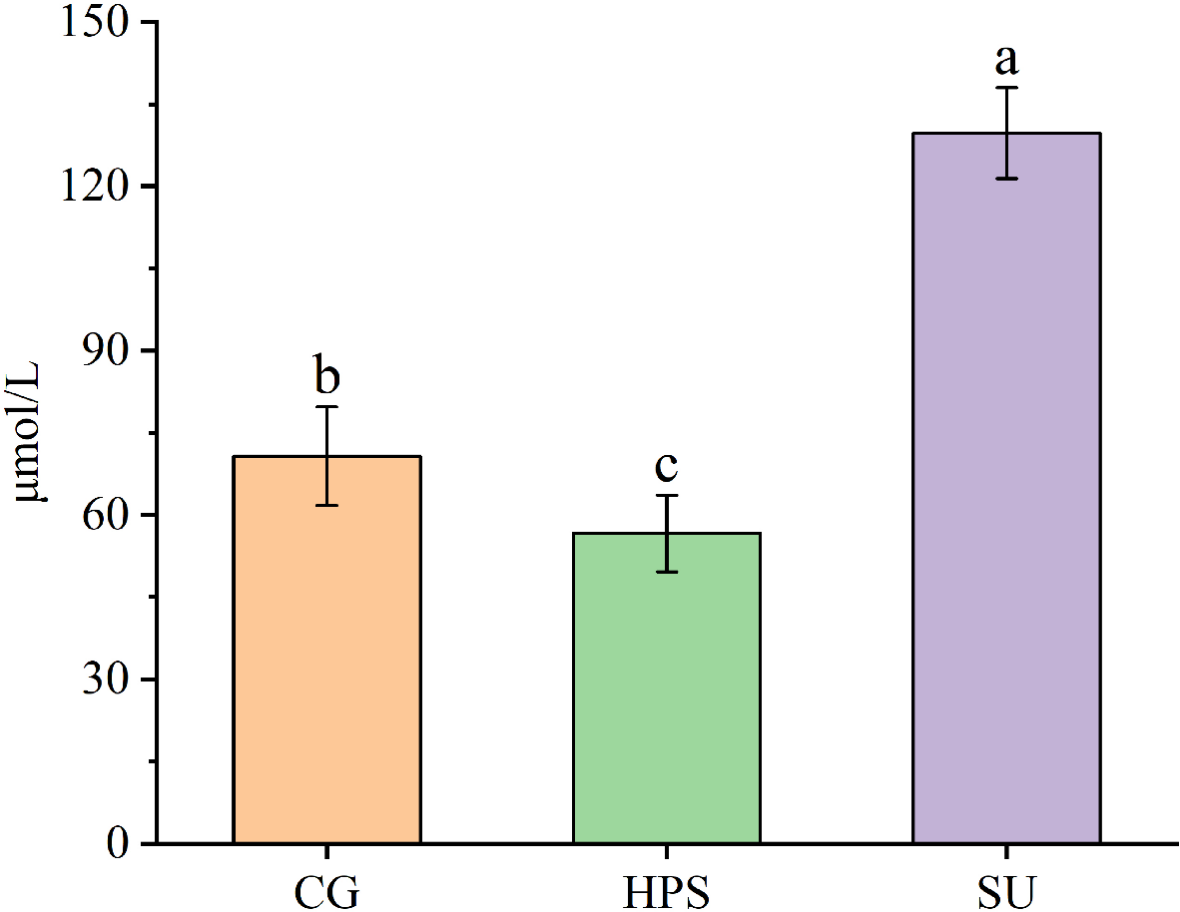
Serum uric acid levels across various treatment groups. Bars marked with the same letter indicate no significant difference (*P* > 0.05).

As shown in Figure 1, the SU group exhibited the highest serum uric acid level at 129.7 µmol/L, which was significantly higher than that of the other two groups (*P* < 0.05). The serum uric acid level for the HPS group was measured at 56.6 µmol/L, which is notably lower than the CG group’s level of 70.8 µmol/L (*P* < 0.05).

### Histological observation of ileum

**Fig. 2:**
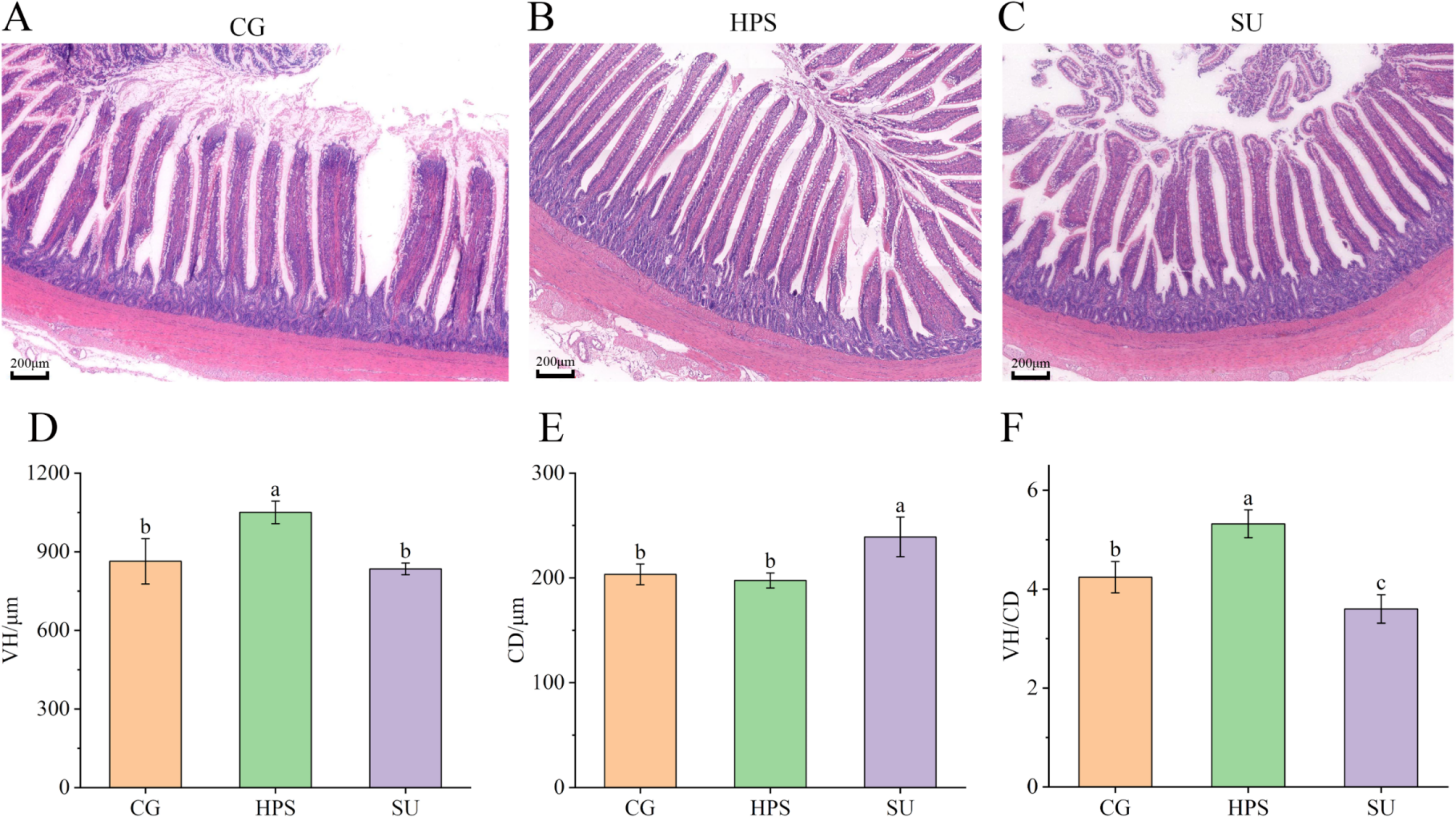
Goose ileum morphological results. (**A–C**) The results of hematoxylin-eosin Staining of the goose ileum in various treatment groups; scale bar: 200 μm. (**D**) Ileal villus. height (VH). (**E**) Ileal crypt depth (CD). (**F**) The ratio of villus height to crypt depth in the ileum (VH/CD). Bars labeled with the same letter indicate no significant difference (*P* > 0.05).

Figure 2 illustrates the histological changes in the ileum across the treatment groups, highlighting key differences. CG group geese exhibit a disordered arrangement of ileal villi and indistinct contours of intestinal epithelial cells (Figure 2A). The ileal tissue structure in the HPS group is intact, with the villi organized orderly (Figure 2B). However, the SU group geese show damaged ileal villi, increased crypt depth, and shedding of intestinal villous epithelium (Figure 2C). At the same time, the villus height (VH) in the HPS group measured 1050.41 µm, which was significantly greater than that in both the CG and SU groups (*P* < 0.05) (Figure 2D). The crypt depth (CD) was 179.62 µm, significantly lower than that in the SU group (*P* < 0.05) (Figure 2E), and the ratio of villus height to crypt depth (VH/CD) was 5.32, significantly higher than that in the CG and SU groups (*P* < 0.05) (Figure 2F).

### Overview of ileal transcriptome data

**Fig. 3:**
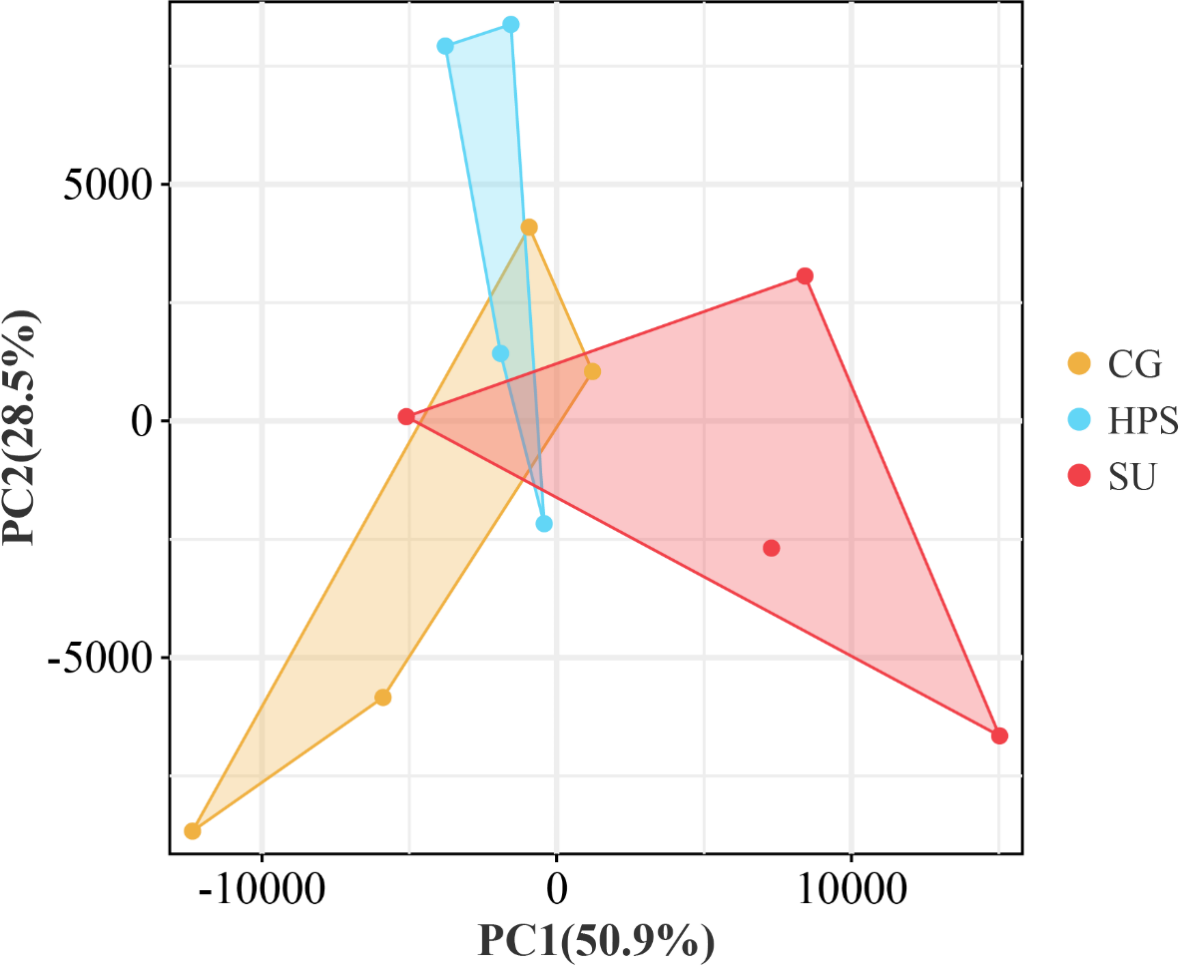
Principal component analysis (PCA) score plot of ileum transcriptomes.

In this experiment, 12 different goose ileum transcriptome libraries were constructed. The average number of raw reads obtained from the experimental samples was 45,337,101. After removing low-quality reads and rRNA from the raw reads, an average of 44,882,192 clean reads was obtained from each sample. The Q30 value ranged from 91.64 to 93.98%, while the unique mapped values from the reference genome ranged from 86.31 to 90.44%, and the total mapped percentages varied from 88.13 to 92.23%. The data is presented in Table 1. Afterward, Principal Component Analysis (PCA) was performed on the 12 samples. We observed that the ileum samples from the CG vs HPS, CG vs SU, and HPS vs SU groups were distributed in different sub-clusters, indicating the reliability of the screened DEGs, as shown in Figure 3.

**Table 1:** Sequencing data statistics.

Sample	Raw reads	Clean reads	Clean Q30	Unique mapped	Multiple mapped	Total mapped
CG1	42,342,038	41,931,266	93.49%	37,026,813 (88.30%)	744,286 (1.78%)	37,771,099 (90.08%)
CG2	42,568,474	42,102,814	93.30%	36,585,615 (86.90%)	811,175 (1.93%)	37,396,790 (88.82%)
CG3	43,576,684	43,174,656	93.05%	37,263,325 (86.31%)	784,973 (1.82%)	38,048,298 (88.13%)
CG4	43,257,164	42,796,956	92.95%	37,413,968 (87.42%)	881,787 (2.06%)	38,295,755 (89.48%)
HPS1	49,514,582	49,167,672	91.87%	44,467,952 (90.44%)	879,020 (1.79%)	45,346,972 (92.23%)
HPS2	48,112,036	47,684,718	91.76%	42,495,236 (89.12%)	878,731 (1.84%)	43,373,967 (90.96%)
HPS3	46,889,378	46,483,146	91.64%	40,687,414 (87.53%)	961,923 (2.07%)	41,649,337 (89.60%)
HPS4	48,547,706	48,092,870	91.76%	42,841,301 (89.08%)	879,127 (1.83%)	43,720,428 (90.91%)
SU1	47,416,448	46,841,920	91.74%	40,445,829 (86.35%)	918,223 (1.96%)	41,364,052 (88.31%)
SU2	47,026,082	46,435,120	92.36%	40,124,874 (86.41%)	896,820 (1.93%)	41,021,694 (88.34%)
SU3	47,739,470	47,275,064	92.78%	41,414,846 (87.60%)	968,146 (2.05%)	42,382,992 (89.65%)
SU4	37,055,144	36,600,104	93.98%	31,903,773 (87.17%)	677,566 (1.85%)	32,581,339 (89.02%)

### Analysis of differentially expressed genes

**Fig. 4:**
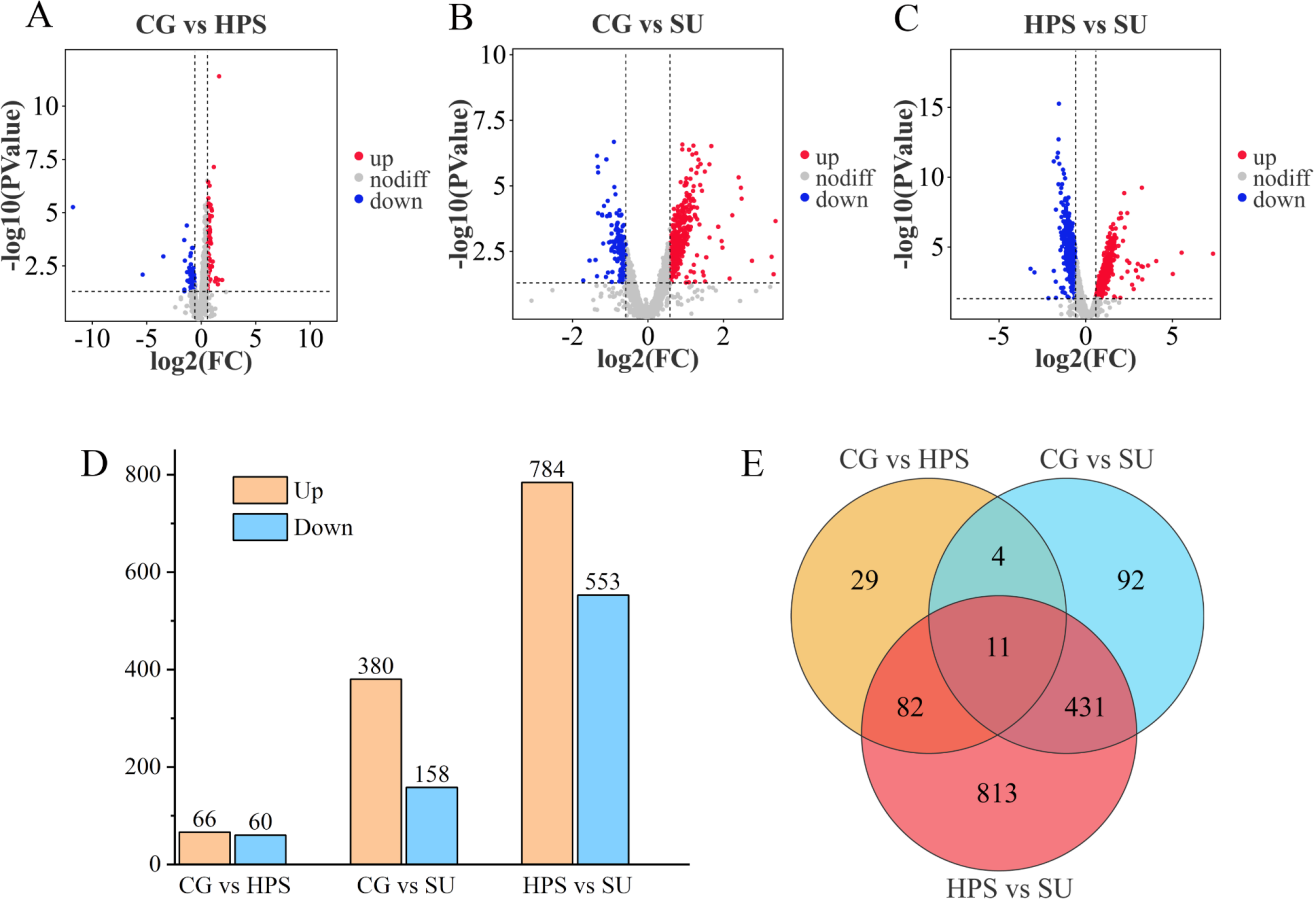
Differentially expressed genes (DEGs) under various treatment groups. (**A–C**) DEGs volcanic maps of CG vs. HPS, CG vs. SU, and HPS vs. SU. The X-axis represents log2(Fold Change) and the Y-axis shows the -log10 (*p*-value). Red dots represent up-regulated genes, blue dots represent down-regulated genes and gray dots represent genes that were not differentially expressed. (**D**) Distribution map of the number of DEGs between the different groups. (**E**) Venn diagram of the DEGs.

For the reliability of the following experimental analysis, the CG, HPS group, and SU groups were compared in pairs, transcriptionally FPKM > 10 in at least one treatment group, |Fold change| ≥ 1.5 between compared groups, and statistical significance at *P* ≤ 0.05. As shown in Figures 4A–D, we identified 126 DEGs (Table S1) in the comparison of CG vs. HPS, which includes 66 up-regulated and 60 down-regulated genes. In the CG vs. SU comparison, 538 DEGs (Table S2) were identified, of which 380 were up-regulated and 158 were down-regulated. For the HPS vs. SU comparison, we identified 1337 DEGs (Table S3; among these, 784 were up-regulated and 553 were down-regulated. We conducted Venn analysis on the three groups of DEGs and found 11 genes in the intersection, as illustrated in Figure 4E.

### GO functional enrichment analysis of differentially expressed genes

**Fig. 5:**
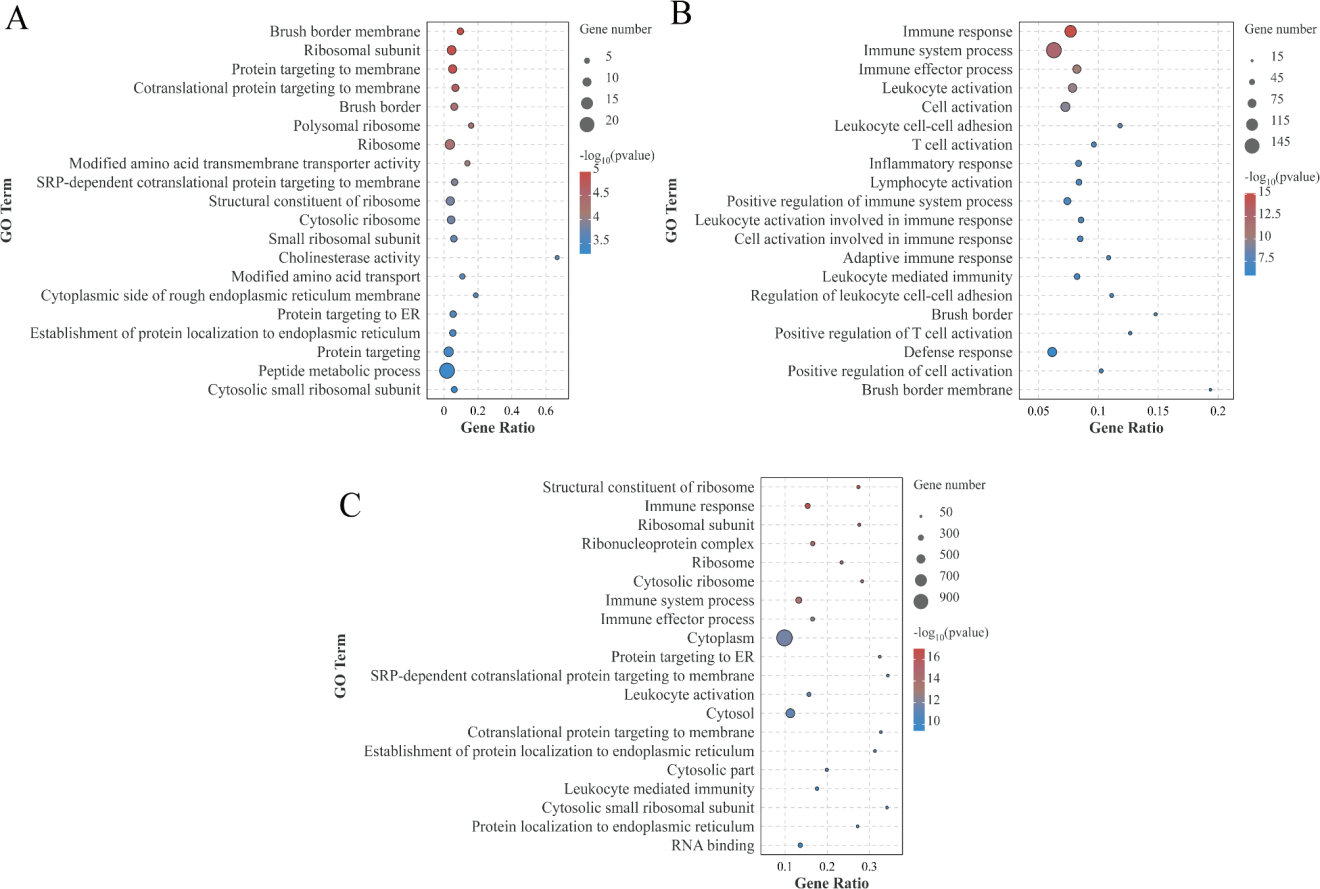
GO enrichment analysis plot for differentially expressed genes (DEGs) across various treatment groups. (**A**) CG vs. HPS. (**B**) CG vs. SU. (**C**) HPS vs. SU. The bubble size represents the number of differential genes enriched in the GO term, and the color represents the enrichment significance in the GO term, and the larger the value, the more significant the enrichment.

We conducted a Gene Ontology (GO) enrichment analysis on the differentially expressed genes (DEGs) identified in the ileum of geese across various treatment groups. The top 20 GO terms are presented in Fig 5 (*P* < 0.05). Among these, the DEGs from the CG vs HPS group showed significant enrichment for 548 GO terms, primarily in Biological Processes and Cellular Components. This includes terms such as Brush border membrane, Ribosomal subunit, Brush border, Protein targeting to membrane, and Cotranslational protein targeting to membrane (Figure 5A). The DEGs in the CG vs SU group were significantly enriched in 1575 GO terms, primarily related to immune functions such as Immune response, Immune system processes, Immune effector processes, Leukocyte activation, and Cell activation (Figure 5B). The DEGs in the HPS vs SU group were significantly enriched in 1758 GO terms, mainly including structural constituents of the Ribosome, Immune response, Cytoplasm, Cytosol, Cytoplasmic parts, and other related terms (Figure 5C).

### KEGG enrichment analysis of differentially expressed genes

**Fig. 6:**
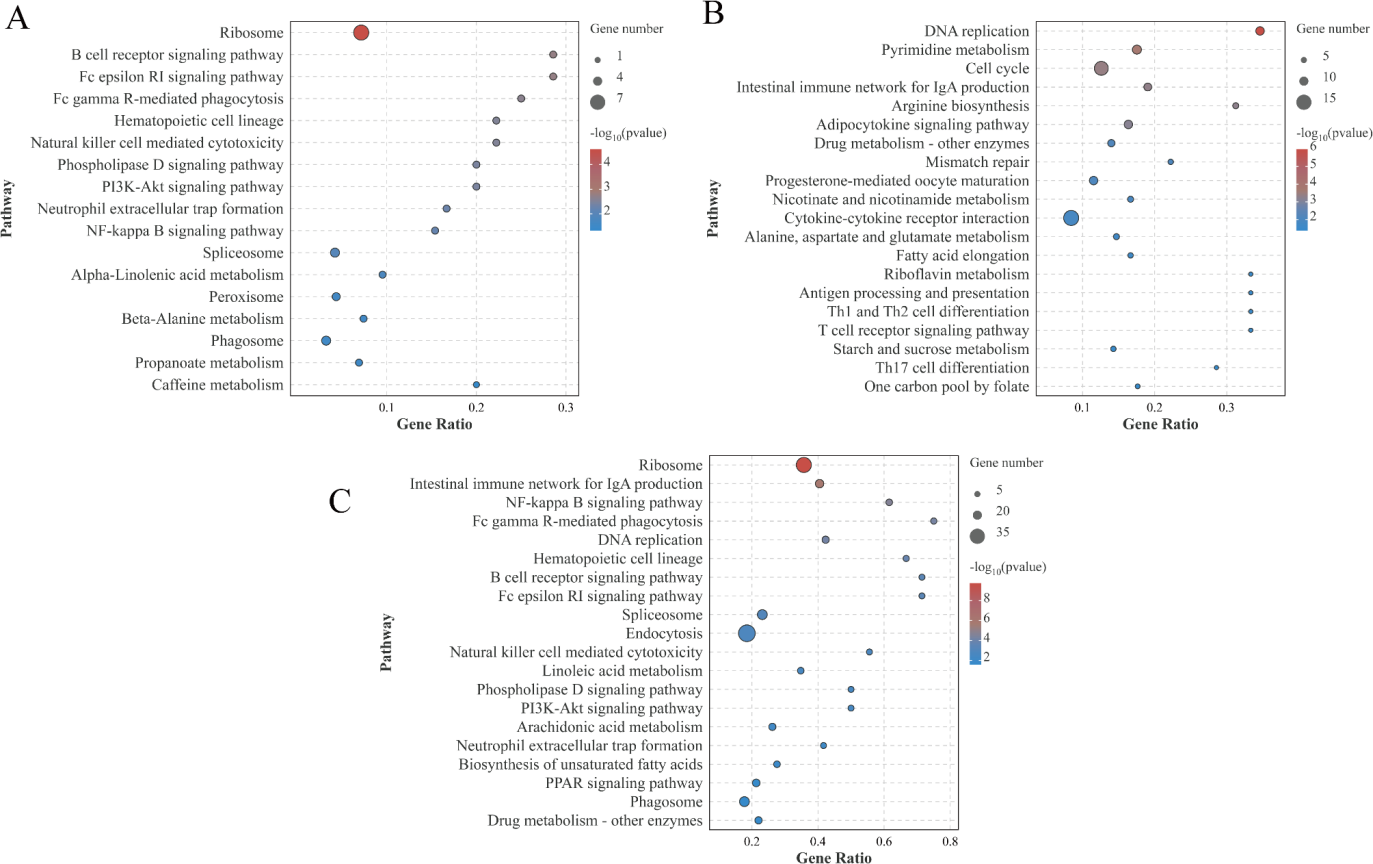
KEGG enrichment analysis plot for differentially expressed genes (DEGs) across various treatment groups. (**A**) CG vs. HPS. (**B**) CG vs. SU. (**C**) HPS vs. SU. The bubble size represents the number of differential genes enriched in the pathway, and the color represents the enrichment significance in the pathway, and the larger the value, the more significant the enrichment.

Next, KEGG enrichment analysis was conducted on DEGs in the ileum of geese across different treatment groups, with the top 20 enriched pathways displayed in Fig 6 (*P* < 0.05). The significantly enriched pathway in the CG vs HPS group was the Ribosome (Figure 6A). The notably enriched pathways in the CG vs SU group were DNA replication, Pyrimidine metabolism, Cell cycle, and the Intestinal immune network for IgA production (Figure 6B). The significantly enriched pathways in the HPS vs SU group included the Ribosome, the Intestinal immune network for IgA production, the NF-kappa B signaling pathway, and the PPAR signaling pathway (Figure 6C).

### Trend analysis

**Fig. 7:**
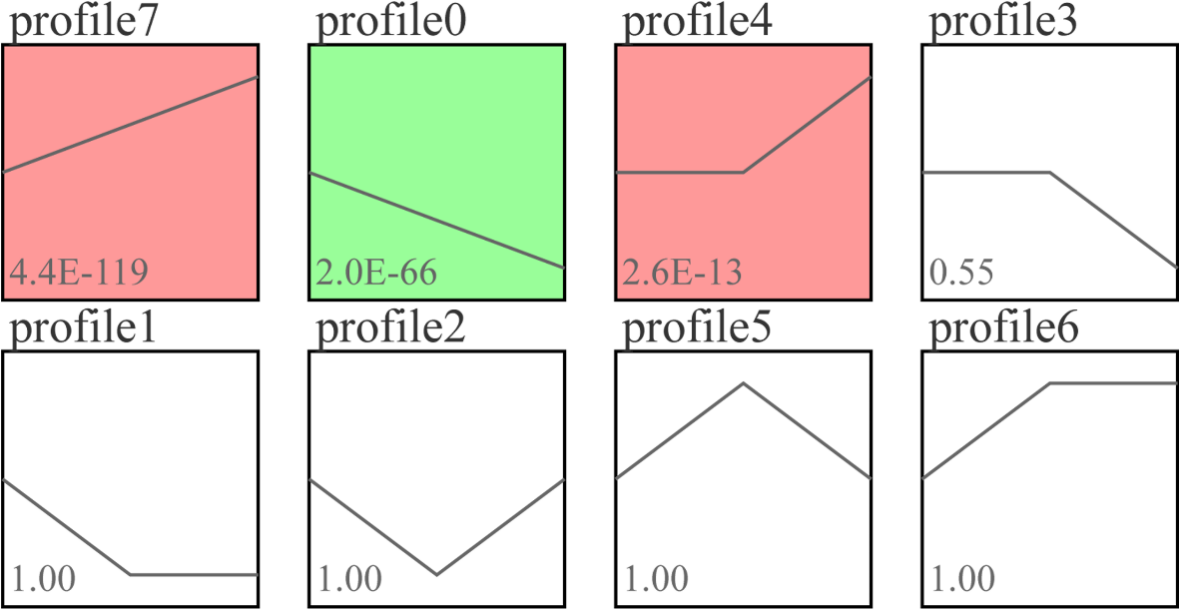
Trend analysis of differentially expressed genes (DEGs). Profile 7 (*n* = 490); Profile 0 (*n* = 395); Profile 4 (*n* = 286). The profile with similar trends has the same color, and the profiles with color have significant enrichment trends (*P* < 0.05). The profiles block without color is a non-significant enrichment trend, and each inflection point is a set of sample data.

**Fig. 8:**
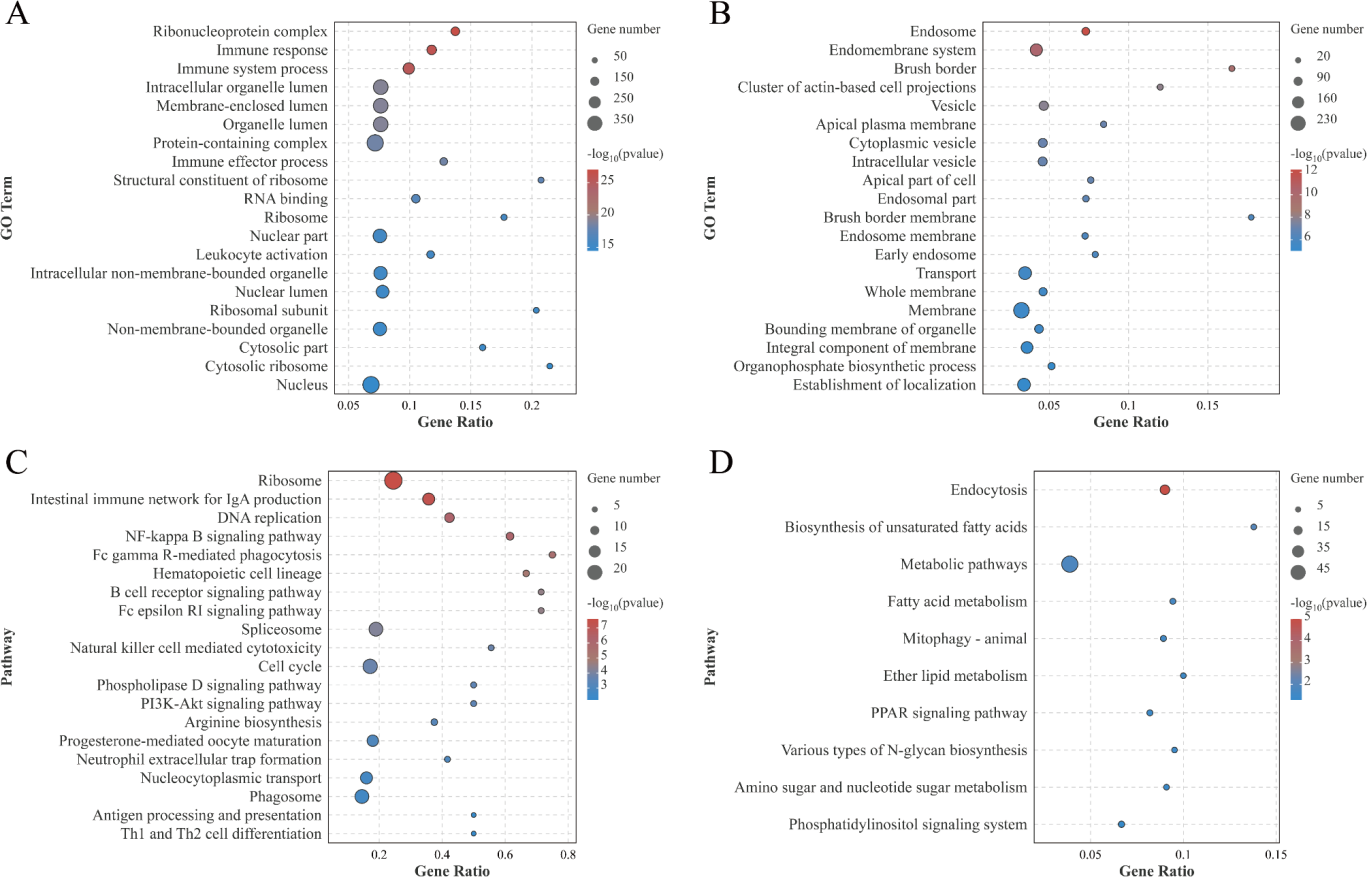
Gene function enrichment analysis of up-profile and down-profile. (**A**) GO enrichment analysis of up-profile; (**B**) GO enrichment analysis of down-profile; (**C**) KEGG enrichment analysis of up-profile; (**D**) KEGG enrichment analysis of down-profile. The bubble size represents the number of differential genes enriched in the GO term (or pathway), and the color represents the enrichment significance in the GO term (or pathway), and the larger the value, the more significant the enrichment.

To reveal the ileum development across different treatment groups, we analyzed the expression trends of differentially expressed genes (DEGs) with *P* < 0.05 as the screening criterion (HPS vs CG vs SU). The differential genes among the three groups were clustered into eight profiles. In Figure 7, the results indicated that three significant gene profiles (profiles 7, 0, and 4) were identified (*P* < 0.05), encompassing a total of 1171 genes. Of these, 776 genes were part of the up-regulated profiles (profiles 7 and 4), while 395 genes were included in the down-regulated profile. GO and KEGG functional enrichment analyses were conducted on the groups of up-regulated and down-regulated gene profiles, with the results depicted in Figure 8. GO functional annotation of up-regulated genes showed significant enrichment in Ribonucleoprotein complex, Immune response, Immune system process, and Intracellular organelle lumen (Figure 8A). KEGG pathway enrichment analysis revealed pathways such as Ribosome, Intestinal immune network for IgA production, DNA replication, and NF-kappa B signaling pathway (Figure 8C). GO functional annotation of down-regulated profile genes was primarily enriched in GO terms related to membrane components such as Endosome, Brush border, Endomembrane system, Membrane, Integral component of membrane, and Membrane part (Figure 8B). Additionally, KEGG pathway enrichment analysis highlighted endocytosis and metabolic pathways (Figure 8D).

### RT-qPCR validation

**Fig. 9:**
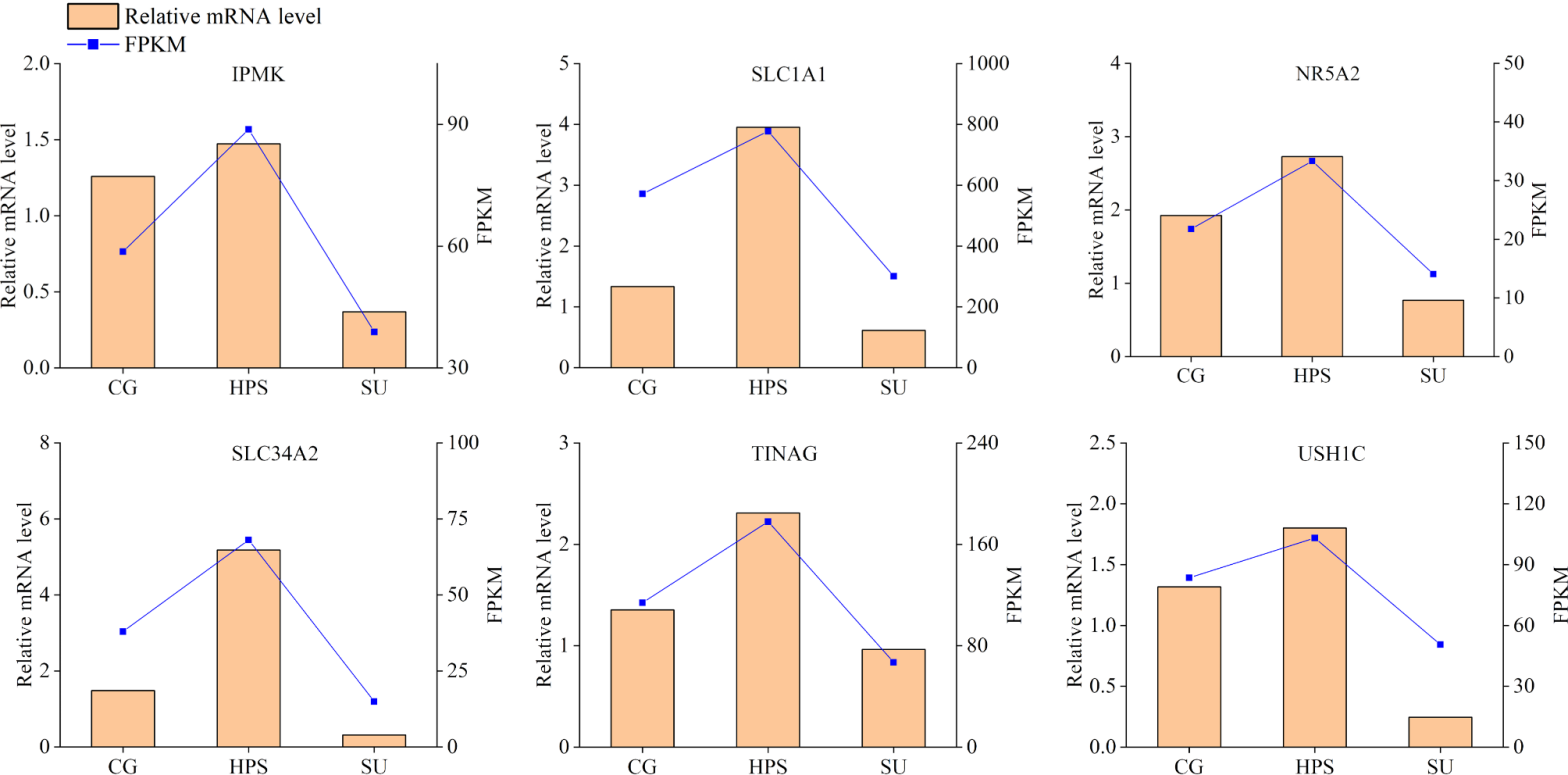
RT-qPCR validation of DEGs. The bar chart illustrates the RT-qPCR results, while the line chart shows the FPKM results from transcriptome sequencing data.

Six genes were randomly selected for RT-qPCR verification. The results, shown in Figure 9, indicate that the expression profiles of these DEGs measured by RT-qPCR were similar to the trends of the RNA-Seq sequencing results, highlighting the reliability of the transcriptome sequencing outcomes.

## Discussions

The small intestine is the most important digestive organ and the largest immune organ. In this experiment, the SU group was administered 30 mg/d of sodium urate to observe the effect of ultra-high concentrations of uric acid on the intestinal health and uric acid metabolism of geese. In previous studies, some researchers have constructed hyperuricemia models through intraperitoneal injection or gavage of uric acid^21,22^. The high-risk period for gout in geese is from 0 to 30 days of age. Goose gout is a metabolic disease caused by several factors, including viruses and bacteria nutrition^1,23^. In this study, 30-day-old healthy geese were chosen as experimental subjects and a high-protein diet was used to induce uric acid metabolism disorders in the geese while excluding other interfering factors.

The results of ileal morphology indicated that VH increased (Figure 2B) and the VH/CD value also increased after feeding HPS (Figure 2F). Interestingly, after feeding SU, VH decreased, CD increased, the VH/CD ratio decreased, and intestinal villi were damaged (Figure 2F). Intestinal health is typically reflected by intestinal VH, CD, and their ratio^24^. Our results indicate that HPS can alleviate damage to the intestinal structure caused by high uric acid levels; however, excessively high concentrations of uric acid will still harm the ileum structure. Research suggests that a diet supplemented with raw potato starch (RPS, a resistant starch) can significantly enhance the villus height (VH) and villus height to crypt depth (VH/CD) ratios of duck cecum^25^. Furthermore, long-term consumption of RPS may improve intestinal morphology and promote epithelial protection in pigs’ colons^26^. In the experiment with rats fed hydroxypropylated corn starch, cecal mucin levels were found to be significantly higher than those in the starch control group^27^. Mucin is a vital component of the intestinal barrier and prevents potential pathogens from entering the underlying epithelium^28^. HPS generates SCFAs through intestinal microbial fermentation, which can lower intestinal pH and promote the growth of beneficial bacteria in the intestine^27^. SCFAs play an essential role in maintaining the normal state of the intestinal epithelium^29^. This may be one reason why HPS improves intestinal morphology.

To reveal critical genes and molecular mechanisms affecting goose intestinal health and uric acid transport, RNA-seq technology was utilized to analyze and compare changes in the transcriptome. The results of GO enrichment analysis indicated that the DEGs identified in the CG vs. HPS group ileum were significantly enriched in membrane components and ribosomes, such as the Brush border membrane, Ribosomal subunit, and Brush border, among others. The brush border is the microvilli-covered surface of the cuboidal and straight-tubed columnar epithelium found in the intestines, where microvilli aid absorption by increasing the surface area of the cells^30^. Transporters positioned at the top of the intestinal villi play a vital role in intestinal absorption and facilitate the transport of nutrients, including carbohydrates, proteins, fats, and SCFAs, within the small intestine^31,32^. Our results indicated that HPS up-regulated the expression of SLC1A1, SLC3A1, SLC5A1, SLC5A12, and SLC5A8 from the Solute Carrier Family. The proteins encoded by SLC1A1 and SLC3A1 act as the primary transporters for intestinal absorption of amino acids^33^. The protein encoded by SLC5A1 plays a crucial role in glucose transport^34^, SLC5A12 and SLC5A8 encode proteins that have the same substrate specificity and are responsible for transporting SCFA. Previous studies have demonstrated that SLC5A12 is expressed in the proximal region of the small intestine of mice and facilitates the transport of SCFAs derived from the diet, whereas SLC5A8 is responsible for transporting SCFAs produced through bacterial fermentation in the distal part of the small intestine^35^, and short-chain fatty acids can reduce serum uric acid in hyperuricemia mice^36^. In the present analysis, HPS up-regulated the expression of SLC5A12 and SLC5A8. It’s important to note that resistant starch primarily produces butyrate and propionate when acted upon by microorganisms^37^. They are the primary substrates for energy metabolism in intestinal cells, and butyrate has been shown to stimulate proliferation at the base of crypts, thus enhancing the renewal and maintenance of intestinal tissue villi^38^. The expression of intestinal SCFA transporters is induced by substrates in response to changes in intestinal SCFA concentration^39^. HPS may enhance the transport of nutrients by the intestinal epithelium by up-regulating the expression of the solute carrier family, thereby indirectly providing a material and energy basis for the growth of intestinal villi. In the SU group, the intestinal CD significantly increased, while the VH/CD significantly decreased. We speculate that the change in intestinal morphology leads to a decrease in nutrient absorption and uric acid excretion, resulting in the accumulation of serum uric acid. Additionally, KEGG enrichment analysis shows that DEGs are primarily enriched in ribosomes, which may help improve intestinal morphology through protein synthesis.

The GO enrichment results for the CG vs SU group indicated that differentially expressed genes (DGEs) were primarily enriched in Immune response, Immune system processes, Immune effect processes, Leukocyte activation, Cell activation, and other immune-related terms. It was found that anti-inflammatory-related genes NFKB1A, LGALS1, and PTPN6^40–42^ were significantly up-regulated in the SU group, suggesting that the intestine was undergoing an inflammatory response. Previous studies have shown that the rise in uric acid in hyperuricemia mouse models is positively correlated with increased intestinal permeability, harmful intestinal bacteria, and damage to the intestinal barrier^43,44^. KEGG enrichment analysis indicated that DGEs were primarily enriched in DNA replication. PCNA plays a role in DNA replication^45^, and its significant up-regulation may indicate intestinal cells are in an active proliferation and differentiation state to resist antigens. The expression of RAC2 in the CG and SU groups was significantly higher than in the HPS group. The protein encoded by RAC2 can participate in the production of reactive oxygen species (ROS), which can directly kill microorganisms and play an important role in host defense^46^. It indicates that HPS can safeguard the intestinal barrier and enhance intestinal health by lowering uric acid levels. This aligns with the morphological findings of this experiment.

The results of GO enrichment analysis in the HPS vs SU group were mainly enriched in immune and cytoplasmic items, and the related DEGs were significantly enriched in the Intestinal immune network for IgA production, NF-kappa B signaling pathway, PPAR signaling pathway, and PI3K-Akt signaling pathway. This study found that the expression of peroxisome proliferator-activated receptor γ (PPARγ), peroxisome proliferator-activated receptor α (PPARα), and PDZ domain-containing 1 (PDZK1) was significantly up-regulated in the HPS group compared to the SU group. IgA is a crucial immunoglobulin in the intestine. As the first barrier, it limits the entry of antigens into the intestinal mucosa, controls the intestinal microflora, and inhibits pro-inflammatory immune response^47^. The pro-inflammatory function of the NF-kappa B signaling pathway has been confirmed in previous studies^48^. PPARγ can reduce inflammation by inhibiting NF-κB, which has a beneficial effect on inflammatory bowel disease^49^, and mice with PPARγ expression defects will develop into ileitis^50^. Studies indicate that PPARα activation is crucial in regulating intestinal cell regeneration, mucosal immunity, and intestinal health permeability^51,52^. This indicates that after uric acid damages the intestinal barrier, microbial invasion triggers an inflammatory response, activates the intestinal immune network, and may participate in the inflammatory response regulated by macrophages and endothelial cells through PPARγ PPARα^53^. In this study, the PI3K-Akt signaling pathway was activated in the HPS group, which preserves epithelial integrity during inflammation^54^. It was found that soluble uric acid increased the expression of PDZK1 and ABCG2 in intestinal cells by activating the PI3K/Akt signaling pathway^55^, and ligand-activated PPARα induced expression of PDZK1^56^. ABCG2 is the primary transporter involved in uric acid excretion in the intestine. It is highly expressed on the apical membrane of intestinal epithelial cells and facilitates the secretion of uric acid from these cells into the intestinal lumen^57^. The results indicated that HPS may lower inflammation and support intestinal health by up-regulating the expression of PPARγ. At the same time, HPS may regulate the expression of PDZK1 by up-regulating PPARα and activating the PI3K-Akt signaling pathway, while also increasing the transport of uric acid in the intestine through the interaction between PDZK1 and ABCG2^55,58^, thereby reducing serum uric acid levels.

Trend analysis was conducted to understand the expression pattern of DGEs. GO and KEGG functional enrichment analysis of up-regulated profiles genes revealed that DEGs were primarily enriched in immune-related processes, including Ribonucleoprotein complex, Immune response, Immune system processes, and the Intestinal immune network for IgA production. The GO and KEGG functional enrichment analysis of down-regulated profile genes demonstrated that DEGs were chiefly enriched in biofilm-related processes such as Endosome, Brush border Endomembrane system, and Metabolic pathways. It indicates that as uric acid concentration increases, the damage to the intestinal structure also worsens, leading to inflammation and stimulating intestinal immunity to produce IgA. Trend analysis indicated that HPS up-regulated the expression of Nuclear Receptor Subfamily 5 Group A Member 2 (NR5A2) and Inositol Polyphosphate Multikinase (IPMK). NR5A2 aids in restoring damaged intestinal epithelium by promoting the proliferation of stem cells and progenitor cells in the intestine crypts^59^. In a mouse model of chronic colitis induced by chemical dextran sulfate sodium (DSS), NR5A2 inhibition was shown to decrease the expression of intestinal steroidogenic genes, lower glucocorticoid synthesis, and sustain chronic inflammation^60^. IPMK can regulate various biological events, mediate the catalytic biosynthesis of inositol polyphosphate, and also regulate key signal transduction factors in a non-catalytic manner. Studies showed that IPMK preserved intestinal integrity by maintaining cluster cell homeostasis in DSS-induced mouse models^61^, and IPMK demonstrated enhanced intestinal epithelial repair function in irinotecan-induced mouse models^62^. Additionally, HPS also up-regulates the expression of ANKS4B and USH1C. The proteins encoded by ANKS4B and USH1C are abundant in brush border microvilli. ANKS4B plays a crucial role in brush border assembly during intestinal epithelial cell differentiation. When USH1C activates ANKS4B and MYO7B, a triplet complex is formed to anchor the microvilli tip to connect cadherin^63,64^. HPS may preserve the integrity of intestinal morphology and promote intestinal health by up-regulating the expression of NR5A2, IPMK, ANKS4B, and USH1C, which play a crucial role in intestinal nutrient absorption and uric acid metabolism.

## Conclusions

This study demonstrates that incorporating HPS positively influences intestinal morphology and uric acid metabolism. This indicates that HPS has considerable potential as a goose feed additive. In the future, gene editing technology may be used to enhance geese’s tolerance to uric acid, thereby reducing the incidence of gout and improving industrial benefits.

## Materials and methods

### Grouping and feeding of geese

In this experiment, 240 25-day-old Yangzhou geese with similar body weights were obtained from Bengbu Huaxin Poultry Co., Ltd., and the experiment commenced after 5 days of adaptive feeding. The 240 Yangzhou geese were randomly divided into three groups: Throughout the study, 5% corn starch was added to the control group (CG) diet, 5% hydroxypropyl starch was included in the hydroxypropyl starch group (HPS) and sodium urate group (SU) diets took 30 mg/d sodium urate on the last 4 days to simulate hyperuricemia. This experiment refers to the research methods of Li and Nagata on hydroxypropyl corn starch and insoluble fibre^27,36^. The experiment lasted for 21 d, with 4 replicates in each group and 20 geese in each replicate. During the experiment, all geese were free to eat and drink water and raised on a net bed. The feed formula is shown in Table 2.

**Table 2:** Composition and nutrient levels of basal diets.

Item	CG	HPS	SU
Ingredient (% of diet)			
Corn	45	45	45
Soybean meal	23	23	23
Wheat grain	9	9	9
Fish meal	7.5	7.5	7.5
Wheat bran	7	7	7
Corn Starch	5	0	0
Hydroxypropyl starch	0	5	5
Limestone	0.7	0.7	0.7
DL-Methionine (98%)	0.3	0.3	0.3
NaCl	1.5	1.5	1.5
Premix^1^	1	1	1
Total	100	100	100
Nutrient levels			
ME (MJ/kg)	11.38	11.16	11.16
Crude protein %	21.13	21.12	21.12
Ether extract %	2.82	2.81	2.81
Crude fibre %	3.30	3.30	3.30
Calcium %	0.66	0.66	0.66
Phosphorus %	0.60	0.60	0.60
Lysine %	1.16	1.16	1.16
Methionine%	0.67	0.67	0.67

^1^The premix provided the following per kg of diets: VD, 1 000 IU VA, 4500I U VE, 30 IU VK3, 1.3 mg VB, 12.2 mg VB2, 10 mg VB12, 1.013 mg VB6, 4 mg Ca, 7.5 mg niacin, 20 mg folic acid, 0.5 mg bio-tin, 0.04 mg Cu, 7.5 mg Fe, 60 mg Zn, 65 mg Mn, 110 mg I, 1.1 mg Se, 0.15 mg.

### Sample collection

Prior to sample collection, the experimental geese were fasted for 12 hours, during which they were allowed to drink water freely. The experimental geese were anesthetized using electric shock, and their blood was collected via the jugular vein method before they were humanely euthanized. The blood was stored at 4 °C for 3 hours to promote blood coagulation. centrifuged at 3000 r/min for 10 min to separate the serum and stored at − 20 °C. The terminal ileum was collected immediately after the goose was sacrificed and fixed in a 4% paraformaldehyde solution. Another terminal ileum was frozen in liquid nitrogen and stored at − 80 °C until RNA isolation. Each replicate randomly slaughtered one goose, and four geese were killed in each group.

## Determination of serum uric acid level

Serum uric acid content was determined by a uric acid detection kit (Nanjing Jiancheng Bioengineering Institute, Nanjing, China). Reaction systems were prepared according to the kit instructions. The reagent mixture, composed of Tris-HCl buffer, peroxidase, and uricase, was added to each reaction system at a volume of 250 µL. For the blank, standards, and measurement systems, 5 µL of distilled water, standard solution, and serum were added, respectively. After thoroughly mixing, the reaction mixtures were incubated at 37 °C for 10 min. Following this, the absorbance of the blank, standards, and measurement systems was recorded at 510 nm and denoted as A_B_, A_S_, and A_M_. The uric acid calibrator (C_C_) concentration was 400 µmol/L, and the uric acid concentration for each sample was calculated using equation (1).

1 $${C}_{UA}=\frac{\left({A}_{M}-{A}_{B}\right)}{({A}_{S}-{A}_{B})}\times {C}_{C}$$

### Histological observation of the ileum

The ileum was initially fixed in a 4% paraformaldehyde solution, which was replaced with a fresh solution after 24 h. Once the tissue was fully fixed, it underwent gradual dehydration using varying concentrations of alcohol, was cleared with xylene, and then embedded in paraffin. The embedded ileum was cut into 5 μm thick sections and stained with hematoxylin and eosin (Biosharp). Finally, the stained sections were observed and photographed using a digital scanning microscope. Image-Pro Plus 6.0 software was employed to examine and analyze the ileum tissue sections of geese, measuring the intestinal villus height and crypt depth.

### RNA extraction, library construction, and sequencing

Total RNA was extracted from the ileum of three groups of geese (n = 12) using a Trizol kit (Invitrogen, Carlsbad, CA, USA). After evaluating RNA integrity on an Agilent 2100 bioanalyzer (Agilent Technologies, Palo Alto, CA, USA), the sequencing library was prepared using Illumina TruSeq mRNA (Illumina, San Diego, CA, USA) following the manufacturer’s instructions and was finally detected with Illumina Novaseq X Plus (Illumina, San Diego, CA, USA).

### Transcriptome alignment and assembly

Raw reads were filtered by fastp (version 0.18.0) and Bowtie2 (version 2.2.8)^65,66^. Paired-end clean reads were mapped to the reference genome using HISAT2 2.1.0^67^. Then the mapped reads of each sample were assembled by using StringTie v1.3.1^68^. Fragment per kilobase of transcript per million mapped reads (FPKM) value was calculated, using RSEM software^69^. The Anser cygnoides reference genome was annotated and downloaded from the GenBank database (GCA_002166845.1).

### Transcriptomic bioinformatics analysis

In order to reveal the relationship between samples, the R package model (http://www.r-project.org/) was used for principal component analysis (PCA). Differentially expressed genes were analyzed by DESeq^70^. The pairwise comparison of CG, HPS, and SU groups was performed. Considering transcriptionally FPKM >10 in at least one treatment group, |Fold change| ≥ 1.5 between compared groups, and statistical significance at *P* ≤ 0.05^71^. Subsequently, gene ontology (GO) and Kyoto Encyclopedia of Genes and Genomes (KEGG) functional enrichment analyses were performed using DESeq2 software. Trend analysis was performed using ShortTime-series Expression Miner software, with |Fold change| ≥ 1.5 as the screening condition, *p*-value < 0.05 was considered statistically significant.

### RT-qPCR analysis

To verify the accuracy of transcriptome sequencing, 6 DEGs were randomly selected for quantitative real-time PCR (RT-qPCR) verification. The reference gene was β-ACTIN, and the primers were designed by Primer Premier 5 (Table 3) and synthesized by Bioengineering (Shanghai) Co., Ltd. The reaction amplification system was 10 µL, which was mainly composed of 5 µL Taq SYBR ® Green qPCR Premix, 0.4 µL forward and reverse primers, 1 µL cDNA template, and 3.6 µL ddH2O. The RT-qPCR amplification procedure follows: pre-denaturation at 95 °C for 5 min, denaturation at the same temperature for 10 s, annealing and extension at 60 °C for 30 s, cycle 45 times. Three biological replicate samples were collected from each sample, and the relative gene expression level was calculated by the 2^−ΔΔCt^ method.

**Table 3:** Quantitative real-time PCR primers.

Gene name	Sequence (5′-3′)		Product length (bp)
IPMK	F	ACCACCTACTGCACCAAATGA	145
	R	GCTGACCTGCTGCTGTATCTT	
SLC1A1	F	AAGTTGAGGACTGGGAAAT	172
	R	ATGAGGGCTGTCAGAAGTG	
NR5A2	F	CTTTAAGCGAACAGTCCAGA	161
	R	CACGCATTCGGTCAGCTC	
SLC34A2	F	CTAACATTGGCACAACCACA	173
	R	CAAACTCTTGCACAACCGTA	
TINAG	F	GCTGGGCACATTACCTCC	124
	R	ATGAATCCACTCAGGCCAT	
USH1C	F	ATGATGTGCTGCGGATGT	120
	R	GGATAAGTGGGCGGATAG	
β-ACTIN	F	CAACGAGCGGTTCAGGTGT	92
	R	TGGAGTTGAAGGTGGTCTCGT	

### Statistical analysis

This study employed a one-way analysis of variance (ANOVA) to assess the differences in uric acid concentration, VH, CD, and VH/CD among the various treatment groups. SPSS 26.0 software was utilized for statistical analysis, and Tukey’s HSD test was employed for post hoc comparison to determine whether the differences between treatment groups were significant. A *p*-value of < 0.05 was regarded as statistically significant. The results were visualized using Origin 2018 software.

## Electronic supplementary material

Below is the link to the electronic supplementary material.

Supplementary Material 1

Supplementary Material 2

Supplementary Material 3

## Data Availability

The original Yangzhou goose ileum sequencing data have been uploaded to the NCBI database, BioProject ID: PRJNA1174150.The BioProject and associated SRA metadata are available at https://dataview.ncbi.nlm.nih.gov/object/PRJNA1174150?reviewer=tser2s96tq8fl7vcl2huprjuv5 in read-only format.
